# Systems Approaches to Predict the Functions of Glycoside Hydrolases during the Life Cycle of *Aspergillus niger* Using Developmental Mutants ∆*brlA* and ∆*flbA*

**DOI:** 10.1371/journal.pone.0116269

**Published:** 2015-01-28

**Authors:** Jolanda M. van Munster, Benjamin M. Nitsche, Michiel Akeroyd, Lubbert Dijkhuizen, Marc J. E. C. van der Maarel, Arthur F. J. Ram

**Affiliations:** 1 Microbial Physiology Research Group, Groningen Biomolecular Sciences and Biotechnology Institute (GBB), University of Groningen, Groningen, the Netherlands; 2 Molecular Microbiology and Biotechnology, Institute of Biology Leiden, Kluyver Centre for Genomics of Industrial Fermentation, Leiden University, Leiden, the Netherlands; 3 Beijerinck Laboratory, DSM Biotechnology Center, Delft, The Netherlands; 4 Aquatic Biotechnology and Bioproduct Engineering Department, Institute for Technology and Management (ITM), University of Groningen, Groningen, the Netherlands; University of Wisconsin—Madison, UNITED STATES

## Abstract

**Background:**

The filamentous fungus *Aspergillus niger* encounters carbon starvation in nature as well as during industrial fermentations. In response, regulatory networks initiate and control autolysis and sporulation. Carbohydrate-active enzymes play an important role in these processes, for example by modifying cell walls during spore cell wall biogenesis or in cell wall degradation connected to autolysis.

**Results:**

In this study, we used developmental mutants (*ΔflbA* and *ΔbrlA*) which are characterized by an aconidial phenotype when grown on a plate, but also in bioreactor-controlled submerged cultivations during carbon starvation. By comparing the transcriptomes, proteomes, enzyme activities and the fungal cell wall compositions of a wild type *A. niger* strain and these developmental mutants during carbon starvation, a global overview of the function of carbohydrate-active enzymes is provided. Seven genes encoding carbohydrate-active enzymes, including *cfcA*, were expressed during starvation in all strains; they may encode enzymes involved in cell wall recycling. Genes expressed in the wild-type during starvation, but not in the developmental mutants are likely involved in conidiogenesis. Eighteen of such genes were identified, including characterized sporulation-specific chitinases and An15g02350, member of the recently identified carbohydrate-active enzyme family AA11. Eight of the eighteen genes were also expressed, independent of FlbA or BrlA, in vegetative mycelium, indicating that they also have a role during vegetative growth. The *ΔflbA* strain had a reduced specific growth rate, an increased chitin content of the cell wall and specific expression of genes that are induced in response to cell wall stress, indicating that integrity of the cell wall of strain *ΔflbA* is reduced.

**Conclusion:**

The combination of the developmental mutants *ΔflbA* and *ΔbrlA* resulted in the identification of enzymes involved in cell wall recycling and sporulation-specific cell wall modification, which contributes to understanding cell wall remodeling mechanisms during development.

## Introduction

The filamentous fungus *Aspergillus niger* is used for the industrial production of enzymes and organic acids, and has been granted a Generally Regarded As Safe status by the US Food and Drug Administration [1,2]. Saprophytic fungi such as *A*. *niger* may encounter nutrient starvation both during industrial fermentations as well as in nature. These conditions induce the expression of hydrolytic enzymes, hyphal fragmentation and loss of biomass, generally referred to as autolysis [3]. Analysis of the transcriptome and proteome of *A*. *niger* and *A*. *nidulans* indicated that carbon starvation activates recycling of cell components and initiates asexual sporulation. Furthermore, these studies identified the up-regulated glycoside hydrolases and proteases [4,5].

During carbon starvation, glycoside hydrolases are thought to degrade the fungal cell wall [3] by acting on its carbohydrate network of β-glucans, chitin, α-glucans, galactomannan and galactosaminogalactan [6]. The role of individual enzymes has been established in a number of cases. For example β-glucanase EngA and chitinase ChiB are responsible for fragmentation of mycelial pellets and a decrease in biomass in carbon-starved cultures of *A*. *nidulans*, and thereby play a role in autolysis [7–9]. Glycoside hydrolases are thus important effectors of cell wall recycling during carbon starvation, thereby generating energy and building blocks that may be used for maintenance and sporulation.

Carbon starvation induces the formation of spore forming structures and spores in submerged cultures of aspergilli [5,10–12]. These morphological changes require extensive remodeling of the fungal cell wall, that can be observed by changes in appearance [10], as well as by the differences in carbohydrate composition of vegetative mycelium and spores [13]. Correspondingly, sporulation specific chitin synthases [14,15] and chitinases [16] have been identified that contribute to these cell wall changes. Thus, glycoside hydrolases and other Carbohydrate-Active enZymes (CAZymes) are also of importance in cell wall remodeling during sporulation.

Understanding the processes initiated by filamentous fungi in response to carbon starvation, such as autolysis and sporulation, is key to understanding an important part of the fungal life cycle, but is also of commercial relevance. Autolytic phenomena may cause problems during industrial fermentations, such as proteolytic product degradation, and in downstream processing [3]. Sporulation affects the accumulation of secondary metabolites in submerged fermentations and has a detrimental influence on the mycelial protein secretion [17,18]. Changes in the fungal cell wall play a role in this reduction in protein secretion [18]. Thus, understanding the regulation of the carbon starvation response and the accompanied expression of carbohydrate-active enzymes as key effectors of cell wall remodeling, is a starting point for manipulation of the carbon starvation response to improve industrial fermentations.

Our understanding of the regulatory mechanisms behind the responses to carbon starvation is incomplete. It involves a complex network of interacting pathways. Asexual sporulation is controlled by the FluG-BrlA regulatory pathway, which has been best investigated in *A*. *nidulans* and is likely to be conserved in *A*. *niger* [10,19]. The cytoplasmic protein FluG synthesizes a small, extracellular, diffusible product that activates the FlbB-E (fluffy low *b* *rlA* expression) protein cascade and subsequently the BrlA transcriptional activator, resulting in asexual sporulation [19,20]. Importantly, in *A*. *nidulans*, a FluG loss of function mutant did not form conidiophores and showed decreased autolysis [21], demonstrating the regulatory connection between asexual sporulation and autolysis. The FlbA protein is a central player in the regulatory pathway, as it connects the cascade of FlbB-E proteins and BrlA to the G-protein coupled receptor signaling that is required to halt vegetative growth during conidiation. FadA is the α-subunit of this heterotrimeric G-protein complex that is further composed of SfaD and GpgA. GTP-bound, activated FadA stimulates growth and blocks sporulation. FlbA activates the GTPase activity of FadA, thus promoting the return of the protein to an inactive state, halting vegetative growth and allowing asexual sporulation [22,23]. Deletion of *flbA* in *A*. *nidulans* results in strongly reduced sporulation and excessive growth of aerial or submerged hyphae followed by autolytic collapse of the mycelium [24,25]. Interestingly, no autolytic phenotype was observed in *A*. *fumigatus* [26]. Very recently, a non-conidiating phenotype has been shown for *flbA* and *brlA* gene deletion strains of *A*. *niger* [18] but so far the effect of *flbA* or *brlA* deletion on autolysis has not been determined.

The expression of glycoside hydrolases and proteases during carbon starvation is one of the factors affecting the autolytic phenotype. In the *A*. *nidulans flbA* deletion strain, complete hyphal disintegration is observed after 3 days of growth in a submerged culture. The chitinase ChiB is required for the observed disintegration, as no such phenotype is observed in a strain carrying both *flbA* and c*hiB* gene deletions [8]. Furthermore, an *A*. *nidulans* strain carrying a deletion of *brlA* was found to have lower expression or delayed up-regulation of *chiB* and *engA* during carbon starvation [7,27]. Similar effects on selected hydrolases or proteases have been found following deletion of other regulators from this pathway [28]. However, a systematic overview of the influence of this regulatory network on the expression of hydrolytic enzymes during carbon starvation is lacking.

Here, we describe the role of glycoside hydrolases during carbon starvation in *A*. *niger* using transcriptomics, proteomics, measurements of enzyme activities and an analysis of their effect on the fungal cell wall. We uncover the set of glycoside hydrolases regulated by the developmental regulators FlbA and BrlA by applying this system-wide analysis to *A*. *niger* wild-type and strains carrying *flbA* or *brlA* gene deletions.

## Methods

### Media and strains

*Aspergillus niger* strains were grown on solidified (2% agar) minimal medium (MM) [29] or complete medium (CM) containing, in addition to MM, 1% yeast extract and 0.5% casamino acids. Minimal medium for bioreactor cultivations was composed as previously described [11] with 8% (w/v) maltose-monohydrate as the sole growth limiting nutrient. The generation of *A*. *niger* strains carrying a *brlA* (An01g10540) or *flbA* (An02g03160) gene deletion in the N402 [30] background, has been described previously [18].

### Bioreactor cultivation and sampling

Except minor modifications regarding the inoculation as described below, duplicate bioreactor batch cultivations were performed as previously described [11] in a 6.6 L Bioflo 3000 bioreactor (New Brunswick Scientific) with a volume of 5 L. Cultures of the non-conidiating ∆*brlA* and ∆*flbA* mutants were inoculated with mycelial biomass obtained from shaking flask pre-cultures grown for 16 h at 30°C in CM at 250 RPM. Prior to inoculation, mycelial biomass was blended and washed twice with minimal medium used for bioreactor cultivation. Cultures were started directly with 750 RPM, sparger aeration (1 L min^-1^) and 0.01% polypropylene glycol as antifoam agent. Throughout cultivation, the pH was maintained at 3.0 by addition of titrants (2 M NaOH or 1 M HCl) and the dissolved oxygen tension (DOT) remained above 40%. Cultivations were sampled at regular intervals as described by [5]. Cultivation and sampling of the wild-type strain N402 has been reported previously [5].

### Transcriptome and proteome analysis

Isolation of RNA, the hybridization to microarrays and data analysis were performed as described by [5]. In short, samples were taken during the exponential growth phase, and after 16 h (day 1), 60 h (day 3) and 140 h (day 6) of carbon depletion of batch fermentations of strains ∆*brlA* and ∆*flbA*. RNA was isolated with the Trizol reagent, cleaned using spin columns and hybridized to Affymetrix whole genome gene chips (Platform GPL6758). Data was analyzed together with those obtained previously for *A*. *niger* wild-type strain N402 [5]. Data normalization was done as described [5], using a False Discovery Rate (FDR) cut-off of 0.005 to determine statistical significance of transcriptional differences. Microarray data of genes encoding CAZymes are available at the Gene Expression Omnibus (GEO) database [31] under accession numbers GSE21752 (strain N402, exponential growth phase) and GSE39559 (strain N402, carbon starvation time points). Data for CAZyme expression in strains ∆*brlA* and ∆*flbA* is available in S1 Table. R/Bioconductor [32] (http://bioconductor.org) was used for the analysis of Affymetrix microarray data. CEL files were processed using the Robust Multi Array Average algorithm [33] as implemented in the affy package [34]. Quality of microarray raw data was tested using various functions provided with the affycoretools package [35]. Differential gene expression analysis was performed using the limma package [36]. Identification of genes with similar expression profiles was guided by hierarchical clustering of genes encoding CAZymes with a minimum normalized expression value of 0.2% of *actA* (An15g00560) and a significant change in expression levels in one or more conditions. Hierarchical clustering analysis was done using McQuitt’s and Ward’s methods as implemented in the hclust function. Heatmaps were generated with a modified version of the aspectHeatmap function of the classdiscovery package [37].

Proteome analysis was performed by LC-MS/MS as described [5] for a single fermentation of the mutant strain ∆*flbA* and compared to the values obtained in duplicate for the wild-type strain [5].

### Detection of enzyme activities

Protease activity in the culture filtrate was measured using N, N-dimethylated BSA as substrate as described previously [5]. Carbohydrate-active enzyme activities in culture filtrates were quantified using isolated fungal cell walls as a model substrate. Cell walls were obtained from *A*. *niger* strain N402, grown in bioreactor batch cultures until the end of exponential growth phase as described above. Mycelium was disrupted using a bead-beater with 425–600 μm glass beads and fragmentation was verified by microscopy. Cell walls were collected by centrifugation, washed and cell wall associated enzyme activity was inactivated by incubating for 10 min at 100°C. Culture filtrates were incubated with 0.1% (w/v) cell walls while shaking at 30°C for 1 h. Reactions were terminated by incubation at 95°C for 15 min. Released carbohydrate monomers were quantified with HPAEC-PAD at least in duplicate for each biological replicate. Monomers were separated on a CarboPac PA1 analytical column at 20°C, using either 100 mM sodium hydroxide as a mobile phase for the separation of N-acetyl-glucosamine and mannose or 12 mM sodium hydroxide to separate glucose and galactose. Detection was performed with a standard quadruple waveform [38].

Exo-acting enzyme activity was separately quantified by incubating 30 μl culture filtrate in 50 μl volume with 0.5 or 1 mM GlcNAc-β-pNP, GalNAc-β-pNP or Glu-β-pNP at 30°C. Reactions were stopped by addition of 0.1 M Na_2_CO_3_, and released pNP was detected at 405 nm. For each biological replicate, at least triplicate measurements were performed for each time point. Statistical analysis was performed using a 2-sided t-test with n = 2.

### Cell wall carbohydrate composition

Mycelium was obtained at 7 time-points during the bioreactor cultivation of wild-type strain N402 and mutant strains ∆*flbA* and ∆*brlA* Mycelium (0.7 g fresh weight) was mixed with 0.5 ml acid washed glass beads (425–600 μm) and disrupted using a mini-bead-beater (Biospec Products). Cell walls were isolated and carbohydrates were hydrolyzed to monomers and quantified by HPAEC-PAD as described in [16]. Changes in the cell wall composition were tested for statistical significance using a two sample t-test.

## Results

### Phenotypic analysis of *brlA* and *flbA* deletion mutants in *A*. *niger*

**Fig 1:**
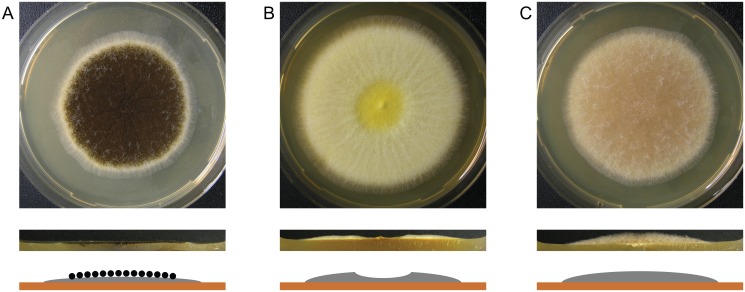
Morphology of N402 (A), ∆*flbA* (B) and ∆*brlA* (C) during growth on agar plates. The wild-type strain N402 forms aerial hyphae and conidiophores with black conidia, clearly visible from the top view (top) and schematic representation (bottom). The mutant strain ∆*flbA* forms only aerial hyphae that collapse in the colony center. Mutant strain ∆*brlA* lacks sporulation and forms conidial stalks that extend into the air, as clearly visible in the side view (middle).

The *A*. *niger* genes An01g10540 and An02g03160 were identified as orthologs of the *A*. *nidulans* developmental regulators *brlA* (AN0973) and *flbA* (AN5893), respectively, and their open reading frames were replaced with the hygromycin resistance cassette in the *A*. *niger* wild-type strain N402 [18]. Both mutants formed aconidial fluffy colonies. Colonies of the ∆*brlA* strain kept developing aerial hyphae that eventually touched the lid of the Petri dish. Aerial hyphae of the ∆*flbA* strain began collapsing in the center of the colony after three days of incubation leading to disintegration of the complete aerial hyphae (Fig. 1). The phenotypes of both deletion strains are in agreement with those described for the *A*. *nidulans* and *A*. *niger* deletion strains [10,18].

### Submerged growth in bioreactor batch cultures

**Fig 2:**
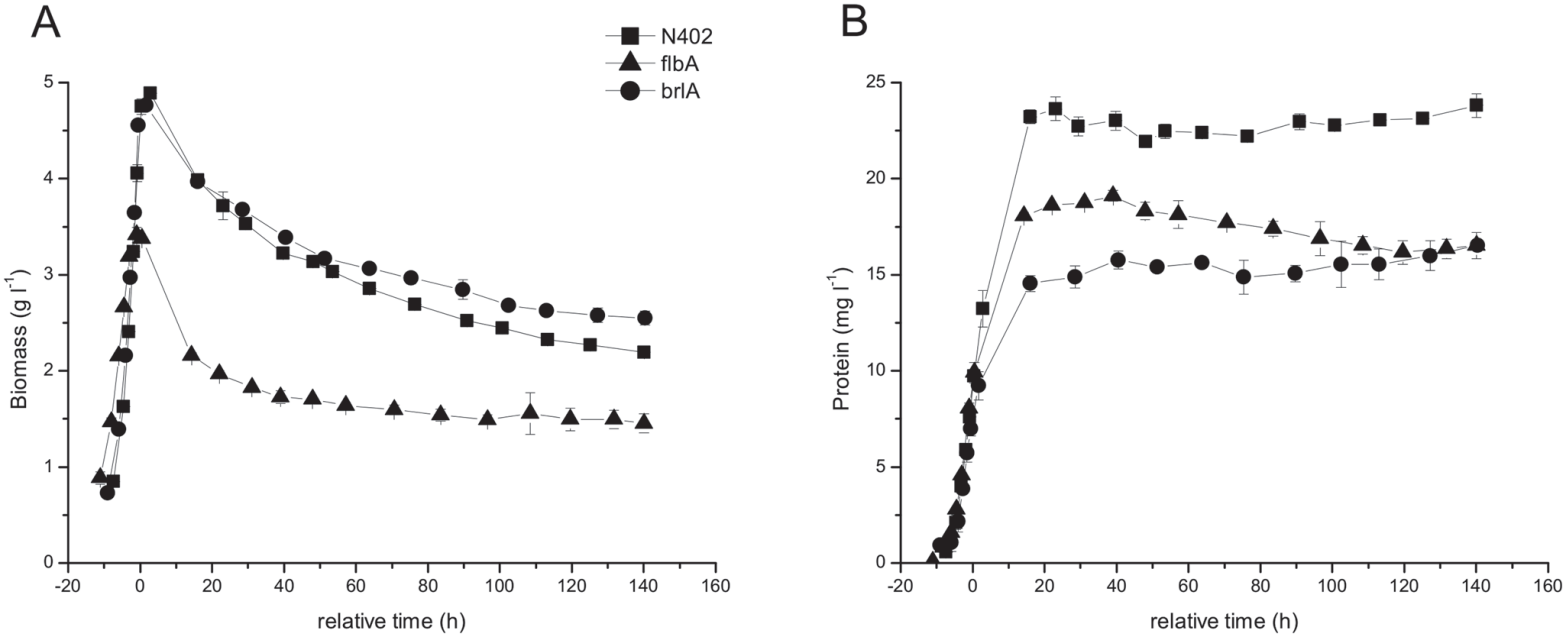
Growth of strains N402 (squares), ∆*flbA* (triangles) and ∆*brlA* (dots) in batch cultures. The biomass (A) in dry weight and protein content of the culture filtrate (B) (Bradford assay), given as mean ± SE of measurements performed for two biological duplicates.

To investigate the properties of the developmental mutants ∆*flbA* and ∆*brlA* during nutrient limitation, the strains were grown in liquid batch cultures in maltose limited minimal medium in a bioreactor and compared to bioreactor cultures of the wild-type strain which have been described previously [5]. Tight control of culture conditions, where the pH was maintained at 3.0, resulted in reproducible biomass profiles and a dispersed mycelial morphology. During exponential growth, biomass accumulation in strains N402 and ∆*brlA* was comparable, with maximum specific growth rates of 0.242 ± 0.001 and 0.218 ± 0.003 h^-1^, respectively. Strain ∆*flbA* grew slower, with a maximum specific growth rate of 0.162 ± 0.006 h^-1^. No differences were observed between the morphology of the wild-type and the ∆*brlA* or ∆*flbA* strains. Growth curves were synchronized at time point t = 0 h, defined as the end of the exponential growth phase, which was recognized by an increase in dissolved oxygen [5]. At the end of the exponential growth phase, the accumulation of biomass in strain ∆*flbA* was with 3.4 ± 0.02 g (kg culture broth)^-1^ less than for the wild-type and ∆*brlA* strains (4.9 ± 0.02 and 4.8 ± 0.01 g (kg culture broth)^-1^, respectively) (Fig. 2A). Upon carbon depletion, a continuous decrease in biomass was observed for strains N402 and ∆*brlA*. During the first 20 h of starvation, biomass decrease was more pronounced for strain ∆*flbA* than for strains N402 and ∆*brlA*, whereas after that only a minimal decrease in biomass was observed for ∆*flbA* (Fig. 2A).

The protein content of culture filtrates (Fig. 2B) increased during exponential growth (up to 0 h) to a concentration of approximately 10 mg l^-1^ for all 3 strains. The protein concentration increased further during the early starvation phase (0–16 h) and remained constant thereafter.

### Transcriptional profiling of genes encoding CAZymes

To identify the CAZymes that require the function of BlrA and FlbA, transcriptional profiles of genes encoding CAZymes were established for the ∆*brlA* and ∆*flbA* strains at four time points during the batch cultivation and compared to the wild-type strain (S1 Table). Samples were taken during exponential growth, and 16 h (day 1), 60 h (day 3) and 140 h (day 6) post carbon depletion, corresponding to time points of the previously published DNA microarray data set for the N402 wild-type strain cultivated under identical conditions [5]. Together, the expression data set consists of 12 different conditions; three strains (wild-type, ∆*brlA* and ∆*flbA*) and four time points. When comparing the transcriptome of genes encoding CAZymes, we observed notable differences between expression of CAZyme-encoding genes over time and in the ∆*brlA* and ∆*flbA* strains, and established groups of genes that shared an expression profile. Based on these expression profiles, we predicted possible functions of several groups of CAZyme-encoding genes, such as a role in cell wall recycling, or in cell wall modification during conidiation. In addition, we found that the expression of only a small number of CAZyme-encoding genes was affected in the *∆brlA* strain, while the effects specific to the *∆flbA* strain were numerous. These findings are presented and discussed in detail in the following paragraphs.

### Genes expressed during starvation in all strains: cell wall recycling

As described previously [5], *cfcA* was one of the genes with the highest increase in transcription after one day of carbon starvation in the wild-type strain (Table 1A). Cfca hydrolyzes cell wall chitin during carbon starvation and is required for fragmentation [39]. Expression of the homologs of this chitinase in *A*. *fumigatus* (*chiB1*) and *A*. *nidulans* (*chiB*) increased during starvation [40,41] and in the latter species the ChiB protein has a role in autolysis and fragmentation [8,9]. Deletion of *flbA* or *brlA* did not appear to affect *cfcA* transcription in *A*. *niger*. In contrast, in *A*.*nidulans* the induction of *chiB* transcription by carbon starvation is delayed in the ∆*brlA* strain [27].

**Table 1:** Normalized gene expression levels of genes discussed in the manuscript.

		wild-type				∆flbA				∆brlA							
ORF	Name	Exp^a^	Day 1	Day 3	Day 6	Exp	Day 1	Day 3	Day 6	Exp	Day 1	Day 3	Day 6	SP^b^	GPI^c^	CAZy family	Function
A																	
An02g07020	CfcA	1.3	69.6	75.9	83.9	2.6	79.2	91.0	57.7	2.0	37.9	73.2	73.6	0	0	GH18	chitobiosidase, group A
An02g11720		2.1	26.0	20.7	22.0	2.0	20.1	18.9	19.5	2.1	14.7	16.4	16.0	0	0	GH38	class 2 ER α-mannosidase
An01g12550		0.8	95.8	85.8	55.5	0.5	101.3	108.5	65.9	0.6	50.3	62.4	42.4	1	0	GH47	α-1,2-mannosidase
An01g06500	DfgD	0.4	2.5	3.6	4.8	0.4	0.7	2.2	3.9	0.4	4.1	7.9	8.7	1	A	GH76	putative α-1,6-mannanase
An07g07700		1.3	27.3	32.6	24.4	1.3	29.1	27.7	20.7	1.1	17.2	16.0	12.7	1	0	GH76	putative α-1,6-mannanase
An04g08730		4.9	18.7	18.2	16.2	2.7	19.4	12.5	16.1	2.0	8.4	9.3	7.3	1	0	GH125	putative exo-α-1,6-mannanase
An14g04240		0.2	4.9	3.1	1.9	0.3	4.3	1.7	0.6	0.2	1.9	2.3	1.5	1	0	GH92	putative α-mannosidase
An11g03120		0.6	10.5	8.6	5.6	0.5	11.6	12.7	9.3	0.5	11.4	8.2	4.8	1	0	GH43	putative endo-β-1,4-xylosidases
An02g10550	AbnC	0.7	48.4	119.5	95.0	0.8	67.5	51.0	36.3	1.7	34.9	26.5	12.4	1	0	GH43	putative endo-α-1,5-arabinanase
An12g01850		0.9	6.2	3.9	5.0	0.8	3.7	3.4	4.7	1.0	6.8	5.7	6.4	0	0	GH2	β-mannosidase
B																	
An01g10540	BrlA	0.3	0.3	11.7	16.4	0.3	0.3	0.3	0.3	0.3	0.2	0.2	0.2	0	0	N/A	transcription factor, role in sporulation
An01g03750	AbaA	0.6	0.5	8.8	20.4	0.6	0.4	0.4	0.3	0.5	0.4	0.6	0.8	0	0	N/A	transcription factor, role in sporulation
An07g03340	Hyp1	1.9	2.6	29.2	72.8	2.0	2.3	3.0	3.3	1.7	2.5	2.8	3.4	1	0	N/A	hydrophobin
An09g05730	FwnA	1.4	0.9	11.4	23.7	0.8	0.7	0.7	0.6	0.7	0.8	0.9	0.7	0	0	N/A	polyketide synthase
An14g05370	BrnA	0.9	0.7	44.1	55.0	0.8	0.6	0.6	0.6	0.5	0.5	0.5	0.5	1	0	N/A	multicopper oxidase
An02g13580	CfcI	0.4	0.8	19.4	38.9	0.3	1.1	0.8	0.6	0.2	0.9	0.3	0.2	1	0	GH18-CBM18	exo-chitinase, group A/C
An09g05920	CtcB	0.3	0.4	11.0	37.0	0.3	0.4	0.4	0.4	0.3	0.5	0.5	0.6	1	0	GH18	endo-chitinase, group B
An15g07370		0.3	0.3	2.2	7.4	0.3	0.3	0.3	0.3	0.3	0.3	0.3	0.3	1	0	CBM14	putative chitin binding module
An06g01530	BgtD	0.8	0.6	2.0	3.9	0.8	1.0	0.9	1.2	0.6	0.5	1.0	1.2	1	0	GH17	putative β-1,3-glucanotransferase
An02g00850		1.2	2.2	5.6	9.8	1.3	1.4	1.0	0.9	1.1	1.2	1.1	1.0	1	1	GH16	endo-β-1,3-glucanase
An02g09050	GelG	0.6	0.6	2.3	5.5	0.8	0.7	0.5	0.6	0.7	0.8	0.7	0.6	1	1	GH72	putative β-1,3-glucanotransferase
An14g05340	UrhgB	0.2	0.3	7.0	8.7	0.2	0.2	0.2	0.2	0.2	0.2	0.2	0.2	1	0	GH105	putative rhamnogalacturonyl hydrolase
An18g01410	DfgA	0.6	0.6	1.3	2.5	0.7	0.6	0.6	0.5	0.7	0.5	0.6	0.6	1	0	GH76	putative α-1,6-mannanase
An06g01140		0.6	0.5	2.3	4.5	0.5	0.4	0.4	0.5	0.5	0.5	0.4	0.4	0	0	GT1	similarity to N-glycosyltransferase ngt–*Saccarothrix aerocolonigenes*
An15g02350		0.9	0.7	51.9	147.0	0.7	0.6	0.5	0.6	0.6	0.6	0.5	0.5	1	0	AA11	putative copper-dependent polysaccharide monooxygenases
C																	
An14g00660	ChsC	5.4	4.6	4.0	4.5	3.4	1.6	0.5	0.5	4.6	2.2	1.3	1.0	0	0	GT2	chitin synthase
An16g02850	CrhF	1.2	0.8	3.7	2.5	3.1	1.1	1.3	1.5	1.2	0.9	1.1	0.9	1	0	GH16	putative chitin-β-1,3-glucan transferase
An10g00400	GelA	9.9	15.7	42.7	42.1	9.8	3.4	2.1	1.3	23.3	15.7	15.8	5.2	1	1	GH72	β-1,3-glucanotransferase
An01g12450	BxgA	7.1	28.0	23.7	36.2	2.9	21.1	3.6	1.2	3.5	10.6	6.3	2.5	1	0	GH55	exo-β-1,3-glucanase
An16g07040	BgtE	48.7	36.1	39.9	30.7	59.9	30.6	11.2	8.8	48.0	24.7	14.2	4.1	1	0	GH17	putative β-1,3-glucanotransferase
An14g02670		0.6	1.3	6.4	3.3	0.8	3.2	0.9	0.8	0.6	0.9	0.9	0.6	1	0	AA9-CBM1	putative copper-dependent polysaccharide monooxygenases
An08g05230		0.5	2.0	12.1	9.3	0.6	5.2	1.1	0.6	0.4	1.2	1.3	0.6	1	0	AA9	putative copper-dependent polysaccharide monooxygenases
An09g05170		0.9	10.0	27.9	32.2	2.2	23.9	12.7	8.4	1.0	8.1	6.1	1.8	1	0	AA11	putative copper-dependent polysaccharide monooxygenases
D																	
An08g09030	CfcB	0.4	0.4	0.6	0.7	0.4	0.4	0.4	0.7	0.3	1.0	5.1	10.6	1	0	GH18	putative chitobiosidase, group A
An14g02180	TpsC	0.5	0.8	1.3	3.5	0.7	1.1	1.6	1.8	0.7	2.1	9.1	21.4	0	0	GT20	putative trehalose-6-phosphate synthase
An02g07770		1.4	3.4	3.6	4.6	3.1	4.1	7.4	8.6	0.8	2.6	9.7	13.5	0	0	GT4	strong similarity to trehalose synthase TSase–*Grifola frondosa*
An13g00400	TppB	0.9	1.2	1.1	1.5	1.2	1.3	1.3	1.3	1.1	2.2	3.5	4.4	1	0	GT20	trehalose-6-phosphate phosphatase
An03g01050		0.4	1.3	0.8	0.7	0.4	0.6	0.6	0.6	0.3	0.9	1.1	4.6	1	0	GH5_16	putative endo-β-1,6-galactanase
An16g03720		1.4	3.0	3.5	5.0	1.2	1.4	2.5	2.3	1.4	3.2	7.5	9.3	0	0	CBM48	putative glycogen binding domain
An16g09090		0.6	0.8	1.7	3.1	0.8	1.1	1.6	2.0	0.7	1.4	3.4	5.3	0	0	GH3	putative β-hexosamidase
E																	
An04g09890	AgsA	1.6	1.3	0.8	0.6	5.5	1.3	0.7	0.6	1.7	0.7	0.5	0.5	1	0	GH13-GT5	α-glucan synthase
An07g07530	CrhB	4.4	3.3	3.6	4.3	21.1	8.1	7.7	7.3	4.8	2.3	6.8	5.9	1	1	CBM18-GH16	putative chitin-β-1,3-glucan transferase
An07g01160	CrhC	2.4	0.9	2.0	2.4	13.6	7.2	4.3	9.0	2.9	1.9	5.6	7.2	1	1	CBM18-GH16	putative chitin-β-1,3-glucan transferase
An03g05530		3.8	2.8	3.2	2.0	18.6	8.2	4.4	3.6	5.1	1.8	1.4	1.0	1	0	GH12	putative endo-β-1,4-glucanase
An16g06800		2.8	1.8	2.6	2.6	27.9	5.5	2.3	2.2	2.7	1.2	1.7	2.0	1	0	GH5_5-CBM1	endo-β-1,4-glucanase
An15g05370	PgaII	0.3	0.6	0.7	0.8	6.7	3.6	0.9	0.5	0.3	0.7	0.4	0.3	1	0	GH28	endo-polygalacturonase
An02g04900	PgaB	2.5	0.7	0.5	0.5	8.5	1.1	0.6	0.6	10.4	1.3	0.6	0.4	1	0	GH28	endo-polygalacturonase
F																	
An02g10310		13.1	12.2	12.9	14.2	2.0	5.8	6.5	5.6	8.0	7.9	6.2	5.6	0	0	GT3	glycogen synthase
An14g04190	GbeA	10.9	42.6	23.7	22.1	2.2	7.2	9.5	8.4	4.1	23.2	21.6	13.3	0	0	CBM48-GH13	glycogen branching enzyme
An02g05260		5.3	17.3	28.8	25.3	0.7	0.7	1.4	1.1	1.8	5.1	10.7	6.1	1	0	CBM21	putative carbohydrate binding module
An09g06400	CtcA	33.1	9.5	6.8	5.5	3.4	0.9	0.4	0.5	16.3	3.8	1.6	0.6	1	1	GH18	endo-chitinase, group B
An08g09610	AgnD	22.4	21.0	21.8	16.2	7.4	4.6	4.7	1.9	38.4	31.9	24.2	12.4	1	0	GH71	putative endo-α-1,3-glucanase
An18g00730		57.2	20.9	3.6	1.6	3.7	1.4	0.9	1.0	59.0	20.2	2.1	0.8	1	0	GT69	
G																	
An09g02240	NagA	0.8	63.6	50.7	49.7	1.5	8.0	10.2	3.7	1.4	38.3	34.9	17.7	1	0	GH20	β-N-acetylhexosaminidase
An01g03090	EngA	0.9	29.9	51.0	52.7	1.2	19.5	18.9	9.3	1.0	16.6	39.8	40.1	1	0	GH81	endo-β-1,3-glucanase
An08g08370		0.3	34.1	31.3	40.0	0.3	8.3	3.2	5.1	0.3	9.0	20.5	31.0	1	0	GH92	putative α-mannosidase
An16g02910		0.5	16.7	9.1	6.6	0.5	2.6	0.5	0.4	0.4	13.3	5.0	2.5	1	0	GH92	putative α-mannosidase
H																	
An09g02160	RgaeA	0.3	5.5	3.3	2.8	0.3	20.5	15.3	8.3	0.3	2.3	1.2	1.7	1	0	CE12	rhamnogalacturonan acetyl esterase
An09g01190	AbnA	0.8	2.5	1.5	1.1	0.8	19.9	21.4	13.2	0.7	1.4	1.1	2.3	1	0	GH43	endo-α-1,5-L-arabinase
An14g01770		1.1	1.7	1.8	1.7	1.0	10.3	4.1	2.6	0.7	1.3	1.3	1.2	1	0	GH3	putative β-glucosidase
An07g09330	CbhA	0.3	0.5	2.0	0.7	0.3	2.5	0.4	0.4	0.3	0.7	0.7	0.7	1	0	GH7	cellobiohydrolase A
An01g11660	CbhB	0.3	0.3	0.4	0.4	0.4	3.2	1.3	0.3	0.3	0.3	0.4	0.3	1	0	GH7-CBM1	cellobiohydrolase B
An15g03550		0.4	0.4	0.8	0.8	0.4	2.0	0.7	0.5	0.4	0.6	0.5	0.3	1	0	GH43	putative α-L-arabinofuranosidase
An03g05260		0.4	0.4	0.4	0.4	0.5	2.8	2.1	1.3	0.4	0.3	0.4	0.3	1	0	GH75	chitosanase
An01g11670		0.2	0.2	0.2	0.2	0.2	1.0	2.6	0.5	0.2	0.2	0.2	0.2	1	0	GH5_5-CBM1	endo-glucanase
An03g00500		1.9	1.9	2.9	2.9	4.4	24.4	15.4	19.7	3.9	2.1	2.9	3.4	1	0	GH30	putative β-1,6-glucanase
An07g08640	AgnB	0.4	29.5	25.9	6.0	0.3	52.0	36.3	51.2	0.3	8.3	10.3	7.6	1	A	GH71	α-1,3-glucanase

Mean gene expression levels given as % of actA expression during exponential growth.

^a^ Exp; exponential growth, Day 1,3 or 6: carbon starvation day 1, 3, 6

^b^ SP: predicted signal peptide presence (1) or absence (0)

^c^ GPI: predicted GPI-anchor presence (1), absence (0) or ambivalence (A)

A number of genes that encode CAZymes with a putative fungal cell wall acting activity share their expression profile with *cfcA*, and thus may also have a function in fungal cell wall recycling. In both wild-type and the ∆*flbA* and ∆*brlA* strains, carbon starvation resulted in increased transcription of the An02g11720 and An01g12550 genes, encoding intracellular α-mannosidase enzymes putatively involved in glycosylation. Also transcription of multiple genes encoding (putative) mannan-acting enzymes increased; the putative GH76 α-1,6-mannanase DfgD that has similarity to enzymes allowing cross-linking of proteins to the fungal cell wall [42,43], the GH76 putative α-1,6-mannanase An07g07700, the GH125 putative exo-α-1,6-mannanase An04g08730 and the GH92 putative α-mannosidase An14g04240. These enzymes form a complete set of activities needed for the break-down and modification of α-1,6-mannan, which is found in the *Aspergillus* cell wall [44].

In all three fungal strains, starvation resulted in increased transcription levels of genes encoding enzymes predicted to be involved in degradation of plant derived carbohydrates. These included the GH43 putative xylosidase encoded by An11g03120, the putative endo-α-1,5-arabinanase encoded by *abnC* and the GH2 β-mannosidase encoded by An12g01850, which could act on substrates such as galacto(gluco)mannan. In view of the substrate specificity of these enzymes, a function in fungal cell wall remodeling during starvation appears unlikely. Alternatively, their expression may be induced by a de-repression mechanism for producing enzymes that scout for alternative carbon sources as proposed by [45–47].

### Genes expressed during prolonged starvation only in the wild-type strain: conidiation

During the carbon-limited batch fermentation of the wild-type strain N402 formation of asexual reproductive structures was observed from day 3 onwards [5]. Coherent with the phenotype of strains ∆*flbA* and ∆*brlA* during growth on agar plates (Fig. 1), conidiation was not observed for these strains in liquid cultures. These differences were reflected in gene transcription levels (Table 1B). For example, transcription of the genes encoding the conidiation-specific regulators BrlA and AbaA (An01g03750), increased 43-fold and 15-fold in wild-type after day 3 of carbon starvation, compared to the exponential growth phase. This increase in transcription was lacking completely in the ∆*flbA* and ∆*brlA* strains. A similar pattern was observed for genes reported to be sporulation related, such as hydrophobin *hyp1/rodA* (An07g03340) [48] as well as polyketide synthase *fwnA* (An09g05730) and multicopper oxidase *brnA* (An14g05370), which are required for the production of spore pigment [49].. Transcription of these genes was increased at least 16-fold, 8-fold and 51-fold respectively only in the wild-type from day 3 of carbon starvation onwards, compared to exponential growth phase. Thus, transcription of conidiation-specific genes was increased from day 3 onwards only in the wild-type.

Seven glycoside hydrolases, a glycoside transferase as well as a protein containing only a CBM14 domain, shared this conidiation-specific expression profile, indicating that their transcription is strictly dependent on the conidiation pathway (Table 1B). Six of these seven up-regulated hydrolases are predicted or known to be active on substrates that are located in the fungal cell wall, indicating that they may have a role in cell wall modification during conidiophore or spore formation. Transcription of the chitinases *cfcI* and *ctcB* was increased most strongly (at least 48-fold and 39-fold respectively) among the conidiation specific hydrolases. CtcB belongs to a phylogenetic group containing only endo-chitinases, and CfcI is an exo-chitinase that hydrolyzes chitin oligosaccharides to monomers [50]. We recently showed that these chitinases are expression in the conidiophores and that they play a role in modification of the cell wall during spore formation by hydrolyzing cell wall chitin [16]. The functional characteristics of these chitinases thus validate our prediction of the role of the genes that share this distinct expression profile.

The gene An15g07370, encoding a protein consisting of a sole carbohydrate binding module of family 14 (CBM14), was expressed together with *cfcI* and *ctcB* in a *brlA* and *flbA* dependent manner, supporting a possible role for this protein in modifying CtcB and CfcI activity during conidiation [16]. In addition, the expression of β-glucan acting BgtD, An02g00850 and GelG during day 3 and day 6 of carbon starvation was strictly dependent on *brlA* and *flbA*. The only glycoside transferase that shared this transcription pattern is the so far uncharacterized GT1 gene An06g01140. Comparative genomics using the *Aspergillus* Genome Database [51] shows that the presence of this gene is restricted to six species, which all belong to the black aspergilli [52].

Compared to other genes in this group, the highest expressed gene was An15g02350. The transcription profile of this gene was highly conidiation-specific: a 60- and 170-fold increase of transcription was observed at day 3 and 6 of carbon starvation in the wild-type strain when compared to exponential growth. No expression was detected in the ∆*brlA* and ∆*flbA* strains. This gene is a member of a CAZy family of copper-dependent lytic polysaccharide monooxygenases that has recently been established, auxiliary activity family AA11. A biochemical function in oxidative cleavage of chitin chains has been demonstrated for the first representative of this family, *A*. *nidulans* AoLpmo11 [53]. Thus, An15g02350 also may be active on chitin, and a function for this enzyme in hydrolysis of cell wall chitin during conidiation would complement the role of the conidiation-specific chitinases CfcI and CtcB in cell wall modification. Importantly, we demonstrated here for the first time a possible physiological role for a member of this AA11 family; fungal cell wall modification during conidiation.

Eight additional carbohydrate active enzymes showed reduced expression during starvation in the ∆*brlA* and ∆*flbA*, while their expression is high in the wild type strain (Table 1C). Different from the group described above is that these genes are also expressed during exponential growth. The expression during exponential growth is not dependant on *flbA* and *brlA*. This pattern suggests that the proteins encoded by these genes have a function during vegetative growth, but also have a function during spore formation. The group of genes with this expression profile included genes encoding cell wall acting enzymes such as chitin synthase ChsC, the putative chitin-β-glucanotransferase CrhF as well as the β-1,3-glucanotransferase GelA, the exo-β-glucanase BxgA and the putative β-glucanotransferase BgtE, which may generate β-1,6-linkages. It also included genes encoding AA9 (previously GH61) and AA11 putative lytic polysaccharide mono-oxygenases.

### Genes up-regulated specifically in ∆*brlA*

Specifically in the ∆*brlA* strain, six genes encoding CAZymes showed ≥ 3-fold increase in transcription levels only after both 3 and 6 days of starvation compared to exponential growth (Table 1D). This increase in transcription levels was absent or much lower in the wild-type or the ∆*flbA* strain. Transcription of *cfcB*, which encodes an enzyme with high sequence similarity to GH18 phylogenetic group A exo-chitinases that release chitobiose, was increased 8- and 15-fold in the ∆*brlA* strain after 3 and 6 days of starvation, respectively.

The disaccharide trehalose (Glc-α-1,1-α-Glc) acts as stress protectant and reserve carbohydrate in fungi. During its synthesis, trehalose-6-phosphate synthase (Tps) produces trehalose-6-phosphate from UDP-glucose and glucose-6-phosphate. Subsequently trehalose-6-phosphate phosphatase (Tpp) removes the phosphate to produce trehalose [54]. Transcription of *tpsC* and gene An02g07770 with similarity to Tps was increased 6-fold and 3-fold respectively in the wild-type on day 6 but much more strongly in the ∆*brlA* strain (13- and 30-fold on day 3 and day 6 respectively for *tpsC* and 12- and 17-fold for An02g07770). Only in the ∆*brlA* strain, transcription of *tppB* was increased 3-fold after 3 and 6 days starvation compared to exponential growth. This suggests that trehalose synthesis may be increased in the ∆*brlA* strain, perhaps signifying a need for increased stress protection.

### Strain ∆*flbA* during the exponential growth phase: signs of cell wall stress

During the exponential growth phase transcription of α-glucan synthase encoding *agsA* was increased 3-fold in ∆*flbA* compared to the wild-type and ∆*brlA* strains (Table 1E). Gene *agsA* is a target of the cell wall integrity (CWI) pathway [55] and this may suggest that strain ∆*flbA* experiences cell wall stress during exponential growth. Accordingly, expression of *crhB* and *crhC*, which encode putative GH16 chitin-β-glucan transferases, increased 5- and 6-fold respectively during exponential growth phase in the ∆*flbA* strain compared to the wild-type strain, and 4- and 5-fold compared to strain ∆*brlA*. The genes c*rhB* and *crhC* have also been shown to be induced in response to caspofungin induced cell wall stress in *A*. *niger* [56], indicating the multiple cell wall integrity pathway target genes are induced in the *flbA* strain. Their *S*. *cerevisiae* homologs *crh1* and *crh2* are up-regulated during cell wall stress [57,58]. These enzymes cross-link chitin to β-glucan, an activity that is part of the mechanism by which the cell counters cell wall stress [59]. The suggestion that ∆*flbA* experiences cell wall stress is further supported by the 135-fold up-regulation of An14g01820 during the exponential growth phase of ∆*flbA* compared to the wild-type; transcription of this gene has been found to be up-regulated in response to cell wall stress (unpublished results, A. Ram).

Surprisingly, expression of three genes predicted to encode plant cell wall degrading enzymes was increased in ∆*flbA* during exponential growth. These included putative endo-β-1,4-glucanases/cellulases belonging to GH12 (An03g05530, 5-fold increase) and GH5 (An16g06800, 10-fold increase) as well as the GH28 endo-polygalacturonase II *pgaII* (23-fold increase). Expression of the gene encoding endo-polygalacturonidase PgaB increased 3- and 4-fold in ∆*flbA* and ∆*brlA* respectively. Together these results indicate that FlbA not only affects gene expression during developmental stage, but that this regulator also has a role during vegetative growth.

### Effect of ∆*flbA* mutation of glycogen metabolism and cell wall modification

We identified 6 genes which showed a low expression in the ∆*flbA* strain during both exponential growth and carbon starvation compared to the wild-type and the ∆*brlA* mutant (Table 1F). Transcriptional changes were observed in 3 genes responsible for glycogen metabolism, which may result in altered turnover of this storage compound. Transcription of the glycogen synthase encoding gene An02g10310 decreased 6-fold during exponential growth of ∆*flbA* compared to the wild-type. Transcription of gene An14g04190, encoding glycogen branching enzyme GbeA, decreased 5- and 6-fold during the exponential growth phase and carbon starvation day 1. Comparable to the regulatory mechanism in higher eukaryotes, the activity of fungal glycogen synthase is regulated by its phosphorylation state [60]. The protein phosphatase-1, which is responsible for dephosphorylating—and thereby activating—glycogen synthase, consists of a catalytic subunit that can interact with different regulatory subunits that modify its function. During dephosphorylation of glycogen synthase in yeast, the regulatory subunit Gac1p interacts with the catalytic subunit Glc7p and through its CBM21 also with glycogen synthase [61]. The *A*. *niger* An02g05260 gene is a homolog of *gac*1. The transcription of this gene strongly decreased in the ∆*flbA* strain, both in the exponential growth phase and during carbon starvation.

Three genes encoding fungal cell wall modifying glycoside hydrolases showed significant changes in expression between the wild-type strain and the ∆*flbA* strain during the exponential growth phase and during carbon starvation. Transcription of the *ctcA* gene decreased around 10-fold in strain ∆*flbA* during the exponential growth phase and during carbon starvation. The encoded chitinase CtcA is an ortholog of the cell wall anchored *A*. *nidulans* ChiA, which is located at hyphal branch sites and at hyphal tips and is thought to be involved in cell wall remodeling during growth [62]. The gene An08g09610, encoding the putative α-1,3-glucanase AgnD, showed a 3- to 8-fold decrease in transcription over the time-course of the ∆*flbA* cultivation. During exponential growth of strain ∆*flbA*, the glycoside transferase An18g00730 was the most repressed CAZyme encoding gene, showing a 16-fold decrease in transcription. It is similarly regulated during day 1 of carbon starvation. One of its homologs in *Cryptococcus neoformans*, Cmt1p, has α-1,3-mannosyltransferase activity [63] while its other homolog, CAP59, is essential for formation of the extracellular protective capsule [64]. This suggests a possible role for An18g00730 in FlbA-dependent synthesis of a cell wall component in *A*. *niger*. The exact role of *flbA* in the expression of these genes remain to be elucidated, but the reduced expression of these putative cell wall biosynthetic genes might relate to reduced integrity of the cell wall which is counteracted by activation of the cell wall remodeling pathway.

### Genes down-regulated in strain *∆flbA* during starvation

The expression of four genes encoding fungal cell wall acting enzymes is at least partly reduced in the *flbA* mutant (Table 1G) during carbon starvation. This includes genes are involved in pellet fragmentation or viability reduction during carbon starvation in *A*. *nidulans*, suggesting a similar role for the genes identified here is possible. During carbon starvation, transcription of the β-N-acetyl-hexosaminidase encoding gene *nagA* increased strongly (≥ 60-fold) in wild-type. The increase in *nagA* expression was strongly reduced in the ∆*flbA* strain. This contrasts with the expression of *cfcA*, the other chitinolytic enzyme encoding gene with a strong increase in transcription during starvation. In the ∆*brlA* strain, the increase of *nagA* transcription was lower than in the wild-type on day 6 of carbon starvation, indicating a partial dependence on this regulator. Deletion of *A*. *nidulans nagA* has been reported to result in an increase in cell viability [9]. The GH81 endo-β-1,3-glucanase EngA [65], in *A*. *nidulans* involved in autolytic fragmentation of mycelia pellets [7] appears to be partly regulated by FlbA, as transcription levels were strongly increased throughout carbon starvation in the wild-type and the ∆*brlA* strain, but less so on day 3 and 6 of carbon starvation in the ∆*flbA* strain.

The gene An08g08370 encodes an enzyme that belongs to GH92, a family of which only bacterial members have been biochemically characterized; most of them were identified as α-mannosidases [66–68]. Transcription of this gene was increased > 90-fold during carbon starvation in the wild-type strain, but only ≥ 11-fold in the ∆*flbA* strain. This increase in transcription was delayed in ∆*brlA* but transcription eventually reached the same level as in the wild-type strain at day 6 of carbon starvation. The gene An16g02910 encodes another GH92 enzyme. Its transcription increased 32-fold in the wild-type strain at day 1 of carbon starvation compared with exponential growth, but only 5-fold in the ∆*flbA* strain.

### Genes up-regulated in strain ∆*flbA* during starvation

The transcription of 10 CAZyme-encoding genes differed between ∆*flbA* on the one hand and the wild-type or ∆*brlA* on the other hand after the transition from exponential growth to day 1 of carbon starvation (Table 1H). The majority of these genes encode CAZymes that degrade plant-derived polysaccharides, most notably the rhamnogalacturonan acetyl esterase RgaeA, endo-1,5-α-L-arabinosidase AbnA and a putative β-glucosidase encoded by An14g01770. RgaeA and AbnA were reported to be part of a set of pectin degrading enzymes that are up-regulated upon carbon source depletion in *A*. *niger* [47]. Also transcription of An03g00500, encoding the putative fungal cell wall-acting β-1,6-glucanase, was increased 3- to 6-fold during carbon starvation in ∆*flbA* but not in the wild-type or ∆*brlA* strains.

The gene AgnB, encoding a putative α-1,3-glucanase had high transcription levels during carbon starvation in the ∆*flbA* strain, while in the wild-type transcription was similarly increased at day 1 and 3 but was decreased at day 6 of carbon starvation.

### Proteome analysis of culture filtrates

Using semi-quantitative proteomics, proteins in the culture filtrate of the ∆*flbA* strain were identified and compared to the previously analyzed proteome of the N402 wild-type culture filtrates [5]. The ∆*flbA* strain was recently reported to have a more complex secretome than the wild-type during growth as sandwiched colony on xylose [18]; at present it is unknown whether this is also the case during submerged fermentations and during carbon starvation.

During carbon starvation of the wild-type and ∆*flbA* strains, the percentage of CAZymes among total proteins was similar with regard to number (29–41%) and amount (mass) (39–53%) of protein. During exponential growth, CAZymes made up 51% of the total amount of detected proteins (24% of total number) in the wild-type strain, but only 22% of the total amount (11% of total number) in the ∆*flbA* strain. This difference was largely due to an increase in intracellular proteins from 15% of the total amount in the wild-type to 41% in the ∆*flbA* strain.

The most abundant CAZymes in the culture filtrates of the wild-type and ∆*flbA* strains were in general similar (Table 2). Glucoamylase GlaA, acid amylase AamA and α-glucosidase AgdA were abundantly present during exponential growth phase and carbon starvation. During carbon starvation in both strains, an increased amount was detected of enzymes that act on cell wall β-glucan, for example for the GH16 chitin-β-glucanotransferase CrhD, GH17 β-glucanotransferase BgtB that generates β-1,6 branches, the β-glucanotransferase GelD and exo-β-1,3-glucanase BxgA. Putative endo-arabinase AbnC and α-arabinofuranosidase AbfB were also secreted at high levels in both strains, despite the low gene expression levels of *abfB*. A noticeable feature in the proteome of both strains was that for a number of cell wall acting enzymes with high transcription levels during carbon starvation, protein was detected only at a low level or not at all. These include the GH81 endo-β-1,3-glucanase EngA, the GH18 chitinase CfcA as well as the GH76 putative α-1,6-mannanase An07g07700.

**Table 2:** Protein levels detected in culture filtrates of the wild-type strain (2 biological replicates) and strain ∆*flbA*.

	Wild-type				Wild-type				∆flbA								
ORF	Exp^a^	Day 1	Day 3	Day 6	Exp	Day 1	Day 3	Day 6	Exp	Day 1	Day 3	Day 6	SP^b^	GPI^c^	CAZy family	Name	Function
An03g06550	6	6	6	6	5	6	6	6	5	6	6	6	1	0	GH15-CBM20	GlaA	glucoamylase
An11g03340	5	6	6	6	5	6	6	5	5	5	6	5	1	0	GH13	AamA	acid α-amylase
An02g10550	4	5	5	5	-	5	6	6	5	5	5	5	1	0	GH43	AbnC	putative endo-α-1,5-arabinanase
An04g06920	4	5	5	5	4	5	5	5	5	5	5	5	1	0	GH31	AgdA	α-glucosidase
An01g11010	3	5	5	5	3	5	6	6*	4	4	5	5	1	1	GH16	CrhD	putative chitin-glucan transferase
An03g05290	4	5	5	5	4	4	5	5	4	5	5	5	1	1	GH17	BgtB	β-1,3-glucanotransferase, β-1,6-branching
An15g02300	2	5	5	5	-	5	5	5	3	5	5	5	1	0	GH54-CBM42	AbfB	α-arabinofuranosidase
An18g03570	2	4	5	5	1	4	5	5	3	4	5	5	1	0	GH3	Bgl1	β-glucosidase
An09g00670	4	5*	5	5*	3	4*	5	5*	4	4	4	5	1	1	GH72-CBM43	GelD	β-1,3-glucanotransferase
An01g12450	2	4	5	5	-	4	5	5	2	3	4	5	1	0	GH55	BxgA	exo-β-1,3-glucanase
An06g00170	2	4	5	5	-	4	5	5*	-	4	4	5	1	0	GH27-CBM13	AglA	α-N-acetylgalactosaminidase
An04g08730	3	4	4	4	2	4	5	5	3	4	5	5	1	0	GH125		putative exo-α-1,6-mannanase
An09g02240	-	4	4	5	-	4	5	5	-	-	-	4	1	0	GH20	NagA	β-N-acetylhexosaminidase
An03g00500	3	4*	4	4	2	4	5	4	4	4	5	5	1	0	GH30		putative β-1,6-glucanase
An01g12550	2	5	4	3	-	5	4	2*	-	3	-	-	1	0	GH47		α-1,2-mannosidase
An03g05530	4	4	4*	4*	4	4*	4	4	4	5	4	4	1	0	GH12		putative endo-β-1,4-glucanase
An16g06800	4	4	4	4	3	4	4	4	5	5	4	3	1	0	GH5-CBM1	EglB	putative endo-glucanase
An11g01540	3	4	4	4	-*	4	4*	4	4	4	4*	4	1	1	GH16	CrhA	putative chitin-glucan transferase
An01g10930	2	4	4	4	2	3	4	4	4	4	4	3	1	0	GH31	AgdB	putative α-glucosidase
An01g04560	-	4	4	4	-	-	4	4	-	-	4	-	1	0	GH16		putative β-1,3-glucanase
An08g03060	2	4	4	4	-	3	4	4	2	-	2	2	1	0	GH92		putative α-mannosidase
An02g11150	2	4*	4*	4	-*	3	4	3	-	4	4	4	1	0	GH27	AglB	α-galactosidase
An09g02160	-	4	4	3	-	4	4	4*	-	4	4	4	1	0	CE12	RgaeA	rhamnogalacturonan acetyl esterase
An12g08280	-	4	4	4	-*	4	4	4	2	3	4*	4	1	0	GH32	InuE	exo-inulinase
An14g04240	-	4*	4	4	-	3	4	4	-	-	2	-	1	0	GH92		putative α-mannosidase
An08g10780	-	3	3	-	-*	3	4	3	-	-*	3	4	1	0	GH43-CBM35	GbgA	putative galactan β-1,3-galactosidase
An03g04190	3	4	3	-	2	4	4	-	3	4	-	-	1	0	EXPN-CBM63		expansin
An02g07020	-	2	-	-	-	2	-	-	-	-	-	-*	0	0	GH18	CfcA	chitobiosidase, group A
An04g06930	2	3	4	4*	2	3	4*	3*	3	3	3*	3	1	0	GH13	AmyC	amylase
An01g01540	3	3	4	4	2	3	4	3	3*	-	3	3	1	0	GH65		α, α-trehalase
An08g05230	-	4	4	-	-	4*	5	-	-	-	-	-*	1	0	AA9		putative copper-dependent lytic polysaccharide monooxygenase
An03g00960	-	3	4	3	-	-	4	-	-	-	-*	-	1	0	GH62	AxhA	β-1,4-arabinoxylan arabinofuranohydrolase
An15g05370	-	-	-	3	-	-	3	3	5	5	5	4	1	0	GH28	PgaII	endo-polygalacturonase
An07g09330	-	-	4	3	-	-	4	4	-	-	-	-	1	0	GH7	CbhA	cellobiohydrolase
An08g08370	-	4	3*	2	-*	3	3	-	-	-	-	-	1	0	GH92		putative ±-mannosidase
An03g01050	-	4*	4	4	-	3*	3	3	-	-	-	-	1	0	GH5		endo-β-1,6-galactanase
An01g00330	-*	3	4	3*	-*	3*	4	3	-	-	3	3	1	0	GH51	AbfA	α-arabinofuranosidase
An11g06540	2	3	4	4	-	-	3*	3	2	2	4	4	1	0	GH2	MndA	β-mannosidase
An09g01190	-	4	4	4	-	-	4	-	-	4	4	-	1	0	GH43	AbnA	arabinan endo-α-1,5-arabinosidase
An14g01800	-*	3	4*	4	-	3	3	3	-	-*	-	3	1	0	GH27		putative α-galactosidase
An14g01790	-	2*	3	3	-*	3*	4	3	-	-	3	3*	0	0	CBM35		puatative galactan binding module
An01g09960	-	3*	4	4*	-	2	3	3	-	-	3	-	1	0	GH3	XlnD	Xylosidase
An07g08640	-	3	2	-	-	3	3	-	-	-	-	-	1	A	GH71	AgnB	α-1,3-glucanase
An14g02670	-	-	4	3	-	-	4*	3	-	-	-	-	1	0	AA9-CBM1		putative copper-dependent lytic polysaccharide monooxygenase
An01g14600	-	-	3	3	-	-	4	-	3	-	-	-	1	0	GH11		putative endo-β-1,4-xylanase
An07g07700	-	3	-	-	-	3	-	-	-	-	-	-	1	0	GH76		putative α-1,6-mannanase
An01g03340	-	-	-	-	-	-	5	-	-	-	-	4	1	0	GH12		xyloglucan-specific endo-β-1,4-glucanase
An10g00400	3	2	-	-	3	2	-	-	3	-	-	-*	1	1	GH72	GelA	β-1,3-glucanotransferase
An04g09650	-	3	3	3	-	-*	3*	2	-	-	-	2	1	1	GH76		putative α-1,6-mannanase
An14g03520	-	3	3	-	-	-	-	-	-	-	-	-	1	1	GH76	DfgC	putative α-1,6-mannanase
An01g01920	-	-	3*	3	-	-	3*	2	-	-	-	-	1	0	GH20		putative β-N-acetylhexosaminidase
An02g13180	-	3	3	3	-	2	3	-	-	-	-	-	1	0	GH55	BgxB	putative β-1,3-glucanase
An01g11660	-	3	3	3	-	-	-	-	-*	-	4	4	1	0	GH7-CBM1	CbhB	cellobiohydrolase
An08g07350	3	3	-	-	2	3	-	-	3	3	-	-	1	1	GH72	GelB	β-1,3-glucanotransferase
An02g04900	-	-	-	-	-	-	-	-	4*	4*	3	3	1	0	GH28	PgaB	endo-polygalacturonase
An11g00200	-	-	-	3	-	-	-	3	-	-	-	-	1	0	GH3		putative β-glucosidase
An07g07530	2	3	-	-	-	3	-	-	4	-	-*	-	1	1	CBM18-GH16	CrhB	chitin-glucan transferase
An16g07040	3	-	-	-	2	3	-	-*	4	3	-	-	1	0	GH17	BgtE	putative β-1,3-glucanotransferase, generating β-1,6 linkages
An09g00260	-	-	-	-	-	-	-	-	-	-	-*	-	1	0	GH36	AglC	α-galactosidase
An09g03260	-	-	-	3	-	-	3	-	-	-	-	3	1	0	GH28	PgaD	endo-polygalacturonase
An11g06080	-	3	-	-	-	3	-	-	-	-	-	-*	1	0	GH3		putative β-glucosidase
An02g00850	-	-	-	2	-	-	-	2	-	-	-	-	1	1	GH16		endo-β-1,3-glucanase
An03g06310	-	-	-	-	-	-	-	-	3*	-*	3	4	1	0	CE8	PmeA	pectin methylesterase
An02g11890	-	2	2	3	-	2	3	3	-	-	-	-	1	0	GH79		β-glucuronidase
An13g02110	-	2	2	2	-	2	3	-	-	-	2	2	0	0	GH29		putative α-L-fucosidase
An01g00780	3	-	-	-	2	3	-	-	3	-	-	-	1	0	GH11	XynB	endo-β-1,4-xylanase
An14g02760	-	-	-	-	-	-	-	-	-*	-	4	4	1	0	GH12	EglA	cellulase
An03g02960	-	-	-	-	-	-	-	-	-	-	3	3	1	0	GH20		putative β-N-acetylhexosaminidase
An09g06400	3	-	-	-	2	-	-	-	3	-	-	-*	1	1	GH18	CtcA	endo-chitinase, group B
An14g04200	-	-	-	-	-	-	3	-	3	-	-	-	1	0	GH28	RhgB	rhamnogalacturonase
An14g01620	-	-	-	-	-	-	3	-	-	-	-	-	1	0	GH79		putative β-glucuronidase
An05g01320	-	-	-	-	-	-	-	-	3*	3*	3	3	1	0	GH5	Man5A	endo-β-1,4-mannanase
An08g01900	-	-	3	2	-*	-	-	-	-	-	-	-	1	0	GH43		putative β-1,4-xylosidase
An16g06990	-	3	-	-	2	-	-	-	2	-	-	3	1	0	GH28	PgaA	endo-polygalacturonase
An08g09610	3	-	-	-	-	-	-	-	3	-	-*	-*	1	0	GH71	AgnD	putative α-1,3-glucanase
An14g04370	-	-	-	-	-	-	-	-	4	-	-	2	1	0	PL1	PelA	pectin lyase A
An04g09700	-	-	-	-	-	-	-	-	-	-	3	3	1	0	GH28	XghA	endo-xylogalacturonan hydrolase
An07g01160	-	-	-	-	-	-	-	-	-	3	3	3	1	1	CBM18-GH16	CrhC	chitin-glucan transferase
An16g02730	-	-	-	-	2	-	-	-	3	-	-	-	1	0	GH43	AbnD	putative endo-arabinanase
An01g06620	-	-	-	-	-	-	-	-	-	-	-	-*	1	0	GH78		putative α-L-rhamnosidase
An16g08090	-	-	-	-	-	-	-	-	-	-	-	3	1	1	GH76	DfgE	putative α-1,6-mannanase
An07g04650	2	-	-	-	-	-	-	-	2	-	-	-*	0	0	GH17	BgtC	putative β-1,3-glucanotransferase generating β-1,6 linkages
An03g05260	-	-	-	-	-	-	-	-	2	-	-	-	1	0	GH75		Chitosanase
An09g03070	1	-	-	-	-	-	-	-	2	-	-	-	1	0	GH13-GT5	AgsE	α—glucan synthase
An16g03720	-	-	-	-	-	-	-	-	2	-	-	-	0	0	CBM48		putative glyogen binding module

Protein levels are quantified as follows: –; not detected, 1; <5 ng ml^-1^, 2;<50 ng ml^-1^, 3;<250 ng ml^-1^, 4: <1000 ng ml^-1^, 5; <4000 ng ml^-1^, 6; >4000 ng ml^-1^. Values marked with an asterix (*) had a relative standard deviation of > 50% between technical replicates.

^a^ Exp; exponential growth, Day 1,3 or 6: carbon starvation day 1, 3, 6

^b^ SP: predicted signal peptide presence (1) or absence (0)

^c^ GPI: predicted GPI-anchor presence (1), absence (0) or ambivalence (A)

A number of differences were found between the extracellular proteins of the wild-type and ∆*flbA* (Table 2). The GH28 endo-polygalacturonase PgaII and GH5 cellulase EglB were detected in increased quantities in ∆*flbA* during exponential growth and carbon starvation, as might be expected from their gene expression levels. The α-1,3-glucanase AgnB was detected in the wild-type during carbon starvation but not in in ∆*flbA*, in contrast to its gene expression levels, that were up-regulated to a similar extent in both strains (day 1 and 3) or up-regulated (day 6) in only ∆*flbA*. The detection of the chitinolytic GH20 β-N-acetyl-glucosaminidase NagA fits with the gene expression profile, the amount of NagA protein in the culture filtrate of ∆*flbA* is reduced compared to that in the wild-type. The GH47 α-1,2-mannosidase An01g12550 as well as GH92 putative α-mannosidases An08g03060, An08g08370 and An14g04240 were detected at lower levels in ∆*flbA* when compared to the wild-type.

### Hydrolytic enzyme activities in culture filtrates

**Fig 3:**
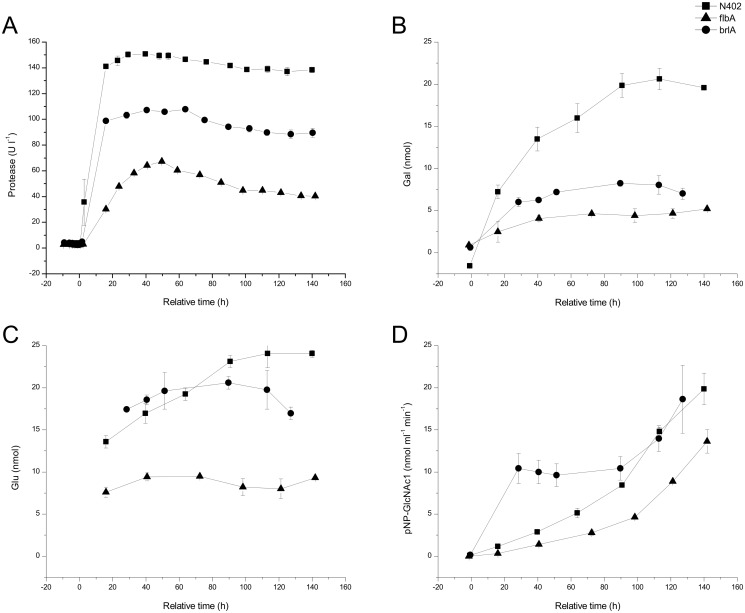
Enzyme activities detected in culture filtrate of batch cultures of strains N402 (squares), ∆*flbA* (triangles) and ∆*brlA* (dots). Protease activity (A), total hydrolytic activity releasing galactose (B) and glucose (C) from the isolated *A*. *niger* cell walls and GH exo-activity releasing N-acetyl-glucosamine from GlcNAc-β-pNP (D). Values are given as mean ± SE of measurements performed for two biological duplicates.

The proteolytic and hydrolytic enzyme activities in the culture filtrates of batch fermentations of the 3 strains was studied. Protease activity (Fig. 3A) increased strongly at the onset of carbon starvation (> 0 h) but subsequently remained stable in time. Throughout the fermentation, proteolytic activity remained lowest for the ∆*flbA* strain.

Carbohydrate hydrolytic capacity in the culture filtrates was determined by assessing the combined activity of endo- and exo-acting enzymes on the carbohydrates in the fungal cell wall. Incubation of fermentation filtrates with isolated fungal cell walls resulted in the release of galactose and glucose. In the wild-type strain enzyme activities releasing galactose (Fig. 3B) and glucose (Fig. 3C) increased during carbon starvation (0–140 h). Compared to the wild-type the culture filtrates of the ∆*brlA* strain had similar glucose releasing activity but lower galactose releasing activity. The culture filtrate of the ∆*flbA* strain contained lower activity in both cases. No release of mannose and N-acetyl-glucosamine from cell walls was detected, indicating that mannanase and chitinase activity was absent. The activity of exo-acting N-acetyl-hexosaminidase was determined separately using the pNP labeled substrate N-acetyl-glucosamine-β-pNP (Fig. 3D). Hydrolysis increased in time throughout starvation, in the wild-type and both developmental mutants. Thus, although no cell wall acting chitinase activity was detected in culture filtrates, exo-chitinolytic activity was present.

### Cell wall carbohydrate composition

**Fig 4:**
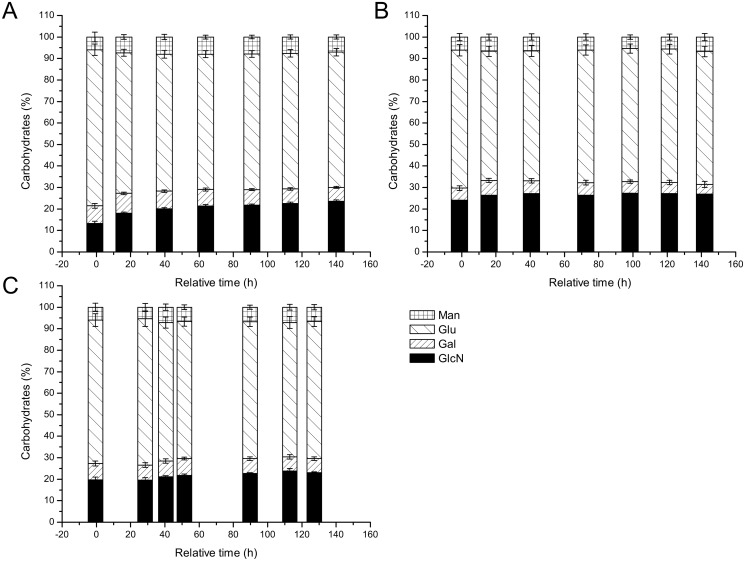
Carbohydrate composition of the cell walls of strains N402 (A), ∆*flbA* (B) and ∆*brlA* (C). Values are given as mean ± SE in % of total moles of carbohydrate as detected in isolated cell walls of two independent growth experiments.

Differential expression and activity of CAZymes and cell wall polymer synthases in strains N402, ∆*flbA* and ∆*brlA* during autolysis and sporulation may result in changes in cell wall composition. Therefore the carbohydrate monomer composition of cell walls was analyzed at seven time points during exponential growth and autolysis. At the end of the exponential growth phase, the total carbohydrates in wild-type strain N402 cell walls were composed of 13% glucosamine, 8% galactose, 73% glucose and 6% mannose (Fig. 4A), consistent with the previously reported composition of *A*. *niger* cell walls [69]. The glucosamine may arise from either N-acetyl-glucosamine or glucosamine, present in the cell wall in chitin or chitosan since the used acid hydrolysis removes the acetyl group from carbohydrates. During carbon starvation, the relative galactose and mannose content remained similar while the glucosamine content increased to 24% and glucose content decreased to 63% at day 6 of carbon starvation.

During the exponential growth phase of ∆*flbA* strain (Fig. 4B), the relative amount of glucosamine detected in cell walls was significantly higher than in wild-type (24 versus 13%), whereas the relative amount of glucose was lower (61 versus 73%). Also small variations in galactose content were observed during carbon starvation but otherwise no significant changes were detected in carbohydrate composition during the course of the batch cultivation. Cell walls of strain ∆*brlA* (Fig. 4C) from the exponential phase contained less glucose (64%) compared to wild-type (73%) and, although with *p* = 0.06 not significant, seemed to contain relatively more glucosamine. During carbon starvation (> 0 h), the monomer composition of the cell walls of this strain was not significantly different from the wild-type.

## Discussion

When filamentous fungi encounter carbon starvation, complex responses are initiated to ensure their survival. Turnover of hyphal biomass by autophagy and cell wall degradation may provide the energy and building blocks required to fuel cellular differentiation and sporulation. Here we present an overview of the physiological effects of the mutational inactivation of the developmental regulators FlbA and BrlA that coordinate the expression of carbohydrate-active enzymes, the key effectors of cell wall remodeling during autolysis and sporulation. Under carefully controlled, reproducible growth conditions, changes in gene transcription, protein secretion, enzyme activities, and their combined effects on the fungal cell wall were investigated.

During growth on solid media, the ∆*brlA* strain shows an a-conidial phenotype as recently described [18]; the mutant strain differentiates to form aerial hyphae and conidial stalks, but does not produce mature conidiophores. Deletion of *flbA* results in inhibition of sporulation and the formation of long aerial hyphae, as described by [18]. In addition, we showed here that these hyphae collapse in the center of the colony, resulting in a phenotype similar to that of an *A*. *nidulans* strain carrying a deletion of *flbA* [25].

During the exponential growth phase in the submerged fermentation, three important differences were identified for ∆*flbA* compared to the wild-type and ∆*brlA* strains. First, the growth rate and the amount of accumulated biomass was lower, in contrast to the lack of differences in biomass levels reported after 7 days of growth as sandwiched colonies on solid media containing xylose [18]. Secondly, the transcriptome data suggest that production of glycogen is reduced in ∆*flbA*, in view of reduced transcription of the glycogen synthase and glycogen branching enzyme encoding genes. In addition, glycogen synthase may be inactivated at the protein level by phosphorylation; the putative regulatory subunit of protein phosphatase 1—that dephosphorylates glycogen synthase—is down-regulated, and deletion of *flbA* may result in continuous activation of protein kinase Pka (similar as reported for *A*. *nidulans*, [70]) which phosphorylates glycogen synthase. Such a reduction in glycogen synthase gene transcription and enzyme activity may be the result of stress experienced during the exponential growth, as described for *Neurospora crassa* [71]. As a result of reduced glycogen synthesis, the ∆*flbA* strain may have accumulated less carbon- and energy reserves to withstand future stress conditions such as carbon starvation. Thirdly, the up-regulated transcription of the CWI pathway target *agsA* and the increased amount of chitin in the cell wall, which may be a result of CWI pathway activation [72], both indicate that the CWI pathway is activated during exponential growth, thus reflecting a requirement for cell wall reinforcement. Indeed the width of the cell wall of strain ∆*flbA* is thinner compared to that of the wild-type [18]. The changes in enzymes (putatively) responsible for cell wall synthesis and modification identified in this study provide a molecular explanation for this finding.

During carbon starvation in the submerged fermentation, *flbA* deletion resulted in a reduction of transcription of the β-N-acetyl-glucosaminidase encoding gene *nagA*, the endo-β-1,3-glucanase encoding gene *engA* as well as genes An16g02910 and An08g08370 that encode GH92 α-mannosidases. Expression levels were increased for β-1,6-glucanase encoding gene An03g00500, the gene encoding α-1,3-glucanase AgnB, and a number of genes encoding putative plant cell wall acting enzymes. The ∆*flbA* strain reportedly has a hyper-autolytic phenotype [25]. In this study this is reflected in a rapid decrease in its biomass during the first day of carbon starvation (Fig. 2A). However, the transcriptome and proteome data of strain ∆*flbA* show that induction of autolytic genes and enzymes was rather limited. Also the enzyme activities detected in the culture filtrates by this strain were lower or equal to those of the wild-type, also giving no indication for hyper-induction of autolytic enzymes. Extracellular GlcNAc-pNP degrading activity was reduced in ∆*flbA*, corresponding with a reduction in *nagA* transcription and NagA protein concentration. It is possible that the enzymes responsible for cell wall degradation are associated with the cell wall and not present in the culture filtrate. However, the amount of biomass and the cell wall composition of strain ∆*flbA* remained unchanged after 1 day of carbon starvation, signifying changes to the wall may be limited. The rapid decrease in biomass during the first day of carbon starvation combined with the lower enzyme activity and lack of cell wall monomer changes indicate that other processes than cell wall degradation may play an important role in the observed autolytic phenotype of the ∆*flba* strain.

The proteomics analysis indicates an increase in the number of total proteins and CAZymes during growth of the ∆*flbA* strain. The *flbA* gene thus is an interesting target for industrial strain improvement. This is in agreement with the previously reported enriched secretome for strain ∆*flbA* grown on solid media [18].

Surprisingly, for a number of genes that had a strongly up-regulated transcription during carbon starvation, such as those encoding chitinase CfcA, β-1,3-glucanase EngA and putative α-1,6-mannanase An07g07700, the corresponding proteins were not detected by the proteome analysis. These enzymes may escape detection by being located in or on the fungal cell wall, or may be unstable under the cultivation conditions. The absence of CfcA, the main chitinase expressed under carbon starvation conditions, and possibly that of other enzymes, may result in incomplete degradation of cell walls during carbon starvation [5]. Indeed, we did not detect endo-chitinase or mannanase activity in culture filtrates of any of the strains under the used cultivation conditions. Furthermore, the composition of the cell wall showed a relative increase of the (N-acetyl-)glucosamine content during carbon starvation, indicating a relative increase in the chitin and/or chitosan content. Together these findings support the conclusion that chitinase, and possibly other activities such as mannanase activity, are required for complete cell wall degradation. Cultivation at pH 3 may lead to inactivation, degradation or increased cell wall association of these enzymes. Preliminary experiments with the wild-type strain showed that when pH control was released upon the onset of carbon starvation, the chitinase CfcA, β-1,3-glucanase EngA and putative α-1,6-mannanase An07g07700 were found in high concentrations in the culture filtrates. During these cultivations the pH increased to 5.8 and microscopy showed increased fragmentation compared to the pH 3 controlled cultivations. We have investigated the action of one of these autolytic enzymes in detail. The pH optimum of chitinase CfcA lies around pH 5, while the activity at pH 3 was reduced to around 50% of the maximum observed activity. Chitinase CfcA hydrolyzed chitin in the cell wall and strongly contributed to cell wall fragmentation in submerged fermentations where the pH control was released at the start of carbon starvation. These results show that under higher pH values, CfcA is present in the fungal culture, the enzyme has higher activity then at low pH values, and its effect on the cell wall can readily be measured and observed [39].

During submerged cultivation of the ∆*brlA* strain, the growth rate, the autolytic degradation of biomass and the cell wall composition were very similar to the wild-type strain. By comparing the ∆*brlA* transcriptome with that of the wild-type and ∆*flbA* strains, we identified a small subset of genes encoding CAZymes that is strictly dependent on BrlA for expression. This strongly suggests that their encoded enzymes are important for the formation of mature conidiophores. All these genes were up-regulated in the aerial structures of sporulating *A*. *niger* colonies growing on agar plates [16]; this shows that their function is conserved under different conditions of sporulation, and is not limited to sporulation in submerged liquid cultures. In addition to the *brlA* dependent CAZymes, a second set of enzymes was identified that are strongly up-regulated during sporulation, but are also expressed under vegetative conditions. These enzymes thus appear to have a role in sporulation but also during other growth conditions. This paper presents the first overview of the *A*. *niger* set of CAZyme-encoding genes that are controlled by the conidiation-specific regulatory pathway. This is of interest to understand the molecular processes underpinning cell wall changes during sporulation. Also, as fungal sporulation requires a significant investment of carbon and energy, manipulation of this process to redirect energy and building blocks to other metabolic routes may allow industrial strain improvement. This may be applicable especially under conditions where *brlA* deletion is not desirable, for example when potential effects on secondary metabolite production need to be avoided. The effect of *brlA* inactivation in *A*. *niger* on secondary metabolite production is still unknown, and although the link between secondary metabolite production and sporulation is often mediated through StuA [73,74], *brlA* inactivation did decrease mycotoxin production in *A*. *fumigatus* [75]. Thus, influencing sporulation by modifying expression of key *brlA* target genes provides an interesting alternative.

## Supporting Information

S1 Table Gene expression levels of genes encoding CAZymes. Mean gene expression levels of all analyzed genes encoding CAZymes, given as % of *actA* during exponential growth.
(XLSX)
